# 2,5-Bis{[(−)-(*S*)-1-(4-bromo­phen­yl)eth­yl]imino­meth­yl}thio­phene

**DOI:** 10.1107/S1600536814003651

**Published:** 2014-02-22

**Authors:** Angel Mendoza, Sylvain Bernès, Guadalupe Hernández-Téllez, Oscar Portillo-Moreno, René Gutiérrez

**Affiliations:** a Centro de Química, Instituto de Ciencias, Benemérita Universidad Autónoma de Puebla, 72570 Puebla, Puebla, Mexico; b DEP, Facultad de Ciencias Químicas, UANL, Guerrero y Progreso S/N, Col. Treviño 64570 Monterrey, NL, Mexico; c Laboratorio de Síntesis de Complejos, Facultad de Ciencias Químicas, Benemérita Universidad Autónoma de Puebla, PO Box 1067, 72001 Puebla, Puebla, Mexico

## Abstract

The title compound, C_22_H_20_Br_2_N_2_S, was synthesized under solvent-free conditions. The mol­ecule shows crystallographic *C*_2_ symmetry, with the S atom of the central thio­phene ring lying on a twofold rotation axis. Furthermore, as a consequence of the (*S*,*S*) stereochemistry, the mol­ecule has a twisted conformation. The dihedral angle between the thio­phene and benzene rings is 72.7 (2)° and that between the two benzene rings is 55.9 (2)°. In the crystal, mol­ecules are arranged in a chevron-like pattern, without any significant inter­molecular inter­actions.

## Related literature   

For the solvent-free organic synthesis, see: Tanaka & Toda (2000 ▶). For the structure of a chiral bis-aldimine compound, see: Espinosa Leija *et al.* (2009 ▶). For structures of thio­phenes substituted in positions 2 and 5 by imine functionalities, see: Bernès *et al.* (2013 ▶); Kudyakova *et al.* (2012 ▶).
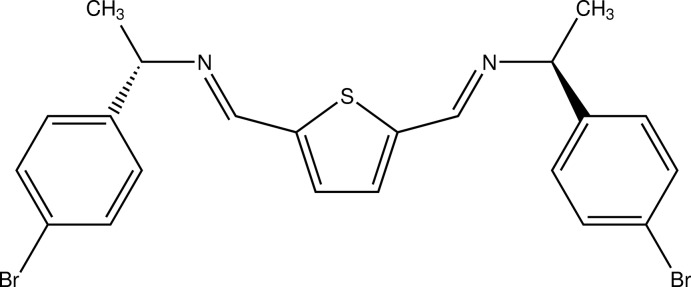


## Experimental   

### 

#### Crystal data  

C_22_H_20_Br_2_N_2_S
*M* *_r_* = 504.27
Monoclinic, 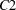

*a* = 24.5329 (15) Å
*b* = 5.9762 (5) Å
*c* = 7.5944 (5) Å
β = 98.536 (6)°
*V* = 1101.11 (14) Å^3^
*Z* = 2
Mo *K*α radiation
μ = 3.79 mm^−1^
*T* = 298 K
0.52 × 0.15 × 0.06 mm

#### Data collection  

Agilent Xcalibur (Atlas, Gemini) diffractometer
Absorption correction: numerical (*CrysAlis PRO*; Agilent, 2013 ▶) *T*_min_ = 0.400, *T*_max_ = 0.815
6101 measured reflections
2107 independent reflections
1630 reflections with *I* > 2σ(*I*)
*R*_int_ = 0.035

#### Refinement  

*R*[*F* ^2^ > 2σ(*F* ^2^)] = 0.036
*wR*(*F* ^2^) = 0.072
*S* = 1.03
2107 reflections
124 parameters
1 restraint
H-atom parameters constrained
Δρ_max_ = 0.20 e Å^−3^
Δρ_min_ = −0.35 e Å^−3^
Absolute structure: Flack (1983 ▶), 914 Friedel pairs
Absolute structure parameter: 0.011 (8)

### 

Data collection: *CrysAlis PRO* (Agilent, 2013 ▶); cell refinement: *CrysAlis PRO*; data reduction: *CrysAlis RED* (Agilent, 2013 ▶); program(s) used to solve structure: *SHELXS2013* (Sheldrick, 2008 ▶); program(s) used to refine structure: *SHELXL2013* (Sheldrick, 2008 ▶); molecular graphics: *ORTEP-3 for Windows* (Farrugia, 2012 ▶); software used to prepare material for publication: *WinGX* (Farrugia, 2012 ▶).

## Supplementary Material

Crystal structure: contains datablock(s) global, I. DOI: 10.1107/S1600536814003651/is5340sup1.cif

Structure factors: contains datablock(s) I. DOI: 10.1107/S1600536814003651/is5340Isup2.hkl

Supporting information file. DOI: 10.1107/S1600536814003651/is5340Isup3.cml

CCDC reference: 987496

Additional supporting information:  crystallographic information; 3D view; checkCIF report

## Figures and Tables

**Fig. 1.**
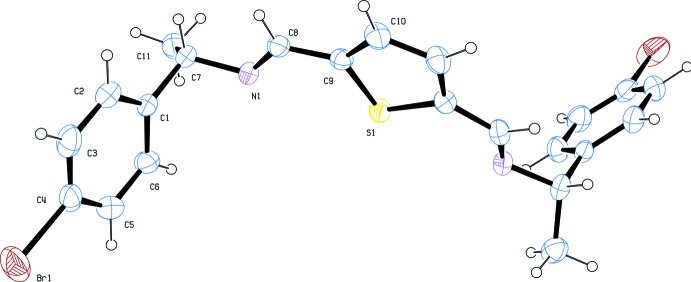
The molecular structure of title compound, with atom labels and 30% probability displacement ellipsoids for non-H atoms.

**Fig. 2.**
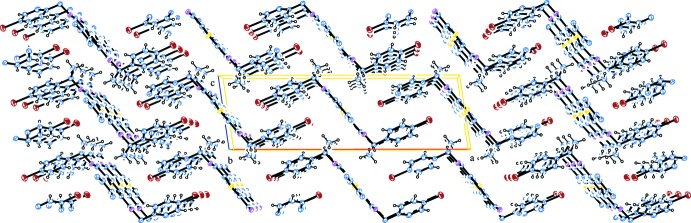
A packing diagram of the title compound, viewed along the b axis.

## supplementary crystallographic information

### 1. Comment

In continuation of our work focused on chiral bisimines (Espinosa Leija *et al.*, 2009; Bernès *et al.*, 2013), we have now synthesized the title compound under the solvent-free approach because of the cleaner, safer, and easier aspects to perform in the synthetic work. So, solvent-free conditions are becoming more popular and it is often claimed that the best solvent is no solvent. (Tanaka & Toda, 2000).

The bis-aldimine compound shows an *E* configuration over every imine bond (Fig. 1). The molecule is an (*S*,*S*) diastereoisomer as expected by synthetic procedure. The asymmetric unit contains a half-molecule. The S atom of the molecule is located on a twofold rotation axis of the space group *C*2. and as a consequence the Br-aldimines groups are placed opposite to the central thiophene ring. Only some previous reports of 2,5-thiophene compounds with achiral or chiral substituents have showed *C*_2_ crystallographic symmetry like title compound (Kudyakova *et al.*, 2012, space group *C*2/*c*; Bernès *et al.*, 2013, space group *P*22_1_2_1_). The crystal packing does not feature any particular interactions (intra or intermolecular) and the supramolecular arrangement in the solid state shows a chevron-like pattern viewed along the *b* axis (Fig. 2).

### 2. Experimental

Under solvent-free conditions, a mixture of 2,5-thiophenedicarboxaldehyde (100 mg, 0.71 mmol) and (*S*)-(–)-1-(4-bromophenyl)ethylamine (285 mg, 1.42 mmol) in a 1:2 molar ratio were mixed at room temperature, giving a white solid. The crude was recrystallized from CH_2_Cl_2_, affording colorless crystals of the title compound.

Yield 96%; m.p. 147–149 °C. Spectroscopic data: [*a*]_D_^25^ = +59.6 (c 1, CHCl_3_). IR (KBr): 1623 cm^-1^ (C=N). ^1^H NMR (400 MHz, CDCl_3_/TMS): δ = 1.51, 1.53 (d, 6H, CHC*H*_3_,), 4.44, 4.45, 4.47, 4.49(q, 2H, C*H*), 7.29–7.45 (m, 10 H, Ar), 8.36 (s, 2 H, *H*C=N). ^13^C NMR (100 MHz, CDCl_3_/TMS) δ = 25.1 (C*C*H_3_), 68.7 (*C*HCH_3_), 120.6 (Ar), 128.3 (Ar), 130.1 (Ar), 131.4 (Ar), 144.0 (Ar), 145.1 (Ar), 152. 7 (H*C*=N). MS—EI: *m/z*= 504 (*M*^+^).

### 3. Refinement

The absolute configuration was determined using the anomalous dispersion of the Br atom, and was that expected from the synthesis, carried-out with an enantiomerically pure amine. All H atoms were placed in calculated positions, with C—H bond lengths fixed to 0.96 Å for the methyl group and 0.93 Å for aromatic CH groups. Isotropic displacement parameters were calculated as *U*_iso_(H) = 1.5*U*_eq_(carrier atom) for the methyl group and *U*_iso_(H) = 1.2*U*_eq_(carrier atom) otherwise.

### Crystal data

**Table:**

C_22_H_20_Br_2_N_2_S	*F*(000) = 504
*M**_r_* = 504.27	*D*_x_ = 1.521 Mg m^−^^3^
Monoclinic, *C*2	Melting point: 420 K
Hall symbol: C 2y	Mo *K*α radiation, λ = 0.71073 Å
*a* = 24.5329 (15) Å	Cell parameters from 1609 reflections
*b* = 5.9762 (5) Å	θ = 4.4–25.2°
*c* = 7.5944 (5) Å	µ = 3.79 mm^−^^1^
β = 98.536 (6)°	*T* = 298 K
*V* = 1101.11 (14) Å^3^	Prism, colourless
*Z* = 2	0.52 × 0.15 × 0.06 mm

### Data collection

**Table:**

Agilent Xcalibur (Atlas, Gemini) diffractometer	2107 independent reflections
Radiation source: Enhance (Mo) X-ray Source	1630 reflections with *I* > 2σ(*I*)
Graphite monochromator	*R*_int_ = 0.035
Detector resolution: 10.5564 pixels mm^-1^	θ_max_ = 26.1°, θ_min_ = 3.0°
ω scans	*h* = −30→30
Absorption correction: numerical (*CrysAlis PRO*; Agilent, 2013)	*k* = −7→7
*T*_min_ = 0.400, *T*_max_ = 0.815	*l* = −9→9
6101 measured reflections	

### Refinement

**Table:**

Refinement on *F*^2^	Hydrogen site location: inferred from neighbouring sites
Least-squares matrix: full	H-atom parameters constrained
*R*[*F*^2^ > 2σ(*F*^2^)] = 0.036	*w* = 1/[σ^2^(*F*_o_^2^) + (0.0288*P*)^2^ + 0.0296*P*] where *P* = (*F*_o_^2^ + 2*F*_c_^2^)/3
*wR*(*F*^2^) = 0.072	(Δ/σ)_max_ < 0.001
*S* = 1.03	Δρ_max_ = 0.20 e Å^−^^3^
2107 reflections	Δρ_min_ = −0.35 e Å^−^^3^
124 parameters	Absolute structure: Flack (1983), 914 Friedel pairs
1 restraint	Absolute structure parameter: 0.011 (8)
0 constraints	

### Special details

**Table:**

Geometry. All e.s.d.'s (except the e.s.d. in the dihedral angle between two l.s. planes) are estimated using the full covariance matrix. The cell e.s.d.'s are taken into account individually in the estimation of e.s.d.'s in distances, angles and torsion angles; correlations between e.s.d.'s in cell parameters are only used when they are defined by crystal symmetry. An approximate (isotropic) treatment of cell e.s.d.'s is used for estimating e.s.d.'s involving l.s. planes.

### Fractional atomic coordinates and isotropic or equivalent isotropic displacement parameters (Å^2^)

**Table:**

	*x*	*y*	*z*	*U*_iso_*/*U*_eq_	
Br1	0.15628 (2)	0.49000 (18)	0.61658 (7)	0.1112 (4)	
S1	0.5	0.2196 (2)	0.5	0.0472 (4)	
C9	0.47486 (16)	0.0181 (7)	0.6292 (5)	0.0427 (10)	
C11	0.4216 (2)	0.4840 (10)	1.0744 (5)	0.0657 (13)	
H11A	0.4194	0.6248	1.0129	0.099*	
H11B	0.4595	0.4499	1.1175	0.099*	
H11C	0.4013	0.4929	1.173	0.099*	
C4	0.2306 (2)	0.4327 (11)	0.7169 (6)	0.0639 (15)	
C1	0.33894 (18)	0.3489 (7)	0.8651 (5)	0.0441 (11)	
C6	0.3243 (2)	0.5418 (9)	0.7670 (6)	0.0569 (13)	
H6	0.3514	0.6457	0.7513	0.068*	
N1	0.43094 (15)	0.2748 (6)	0.8052 (5)	0.0452 (9)	
C5	0.2703 (2)	0.5823 (9)	0.6921 (7)	0.0674 (16)	
H5	0.2613	0.711	0.6253	0.081*	
C2	0.2971 (2)	0.1997 (9)	0.8837 (6)	0.0603 (13)	
H2	0.3056	0.0688	0.9482	0.072*	
C3	0.2431 (2)	0.2389 (11)	0.8092 (7)	0.0690 (14)	
H3	0.2157	0.135	0.8218	0.083*	
C7	0.3974 (2)	0.3024 (8)	0.9485 (5)	0.0501 (12)	
H7	0.398	0.1616	1.0149	0.06*	
C8	0.44434 (18)	0.0769 (7)	0.7709 (6)	0.0469 (12)	
H8	0.4339	−0.038	0.8414	0.056*	
C10	0.4853 (2)	−0.1907 (8)	0.5728 (7)	0.0540 (13)	
H10	0.4743	−0.3205	0.625	0.065*	

### Atomic displacement parameters (Å^2^)

**Table:**

	*U*^11^	*U*^22^	*U*^33^	*U*^12^	*U*^13^	*U*^23^
Br1	0.0529 (4)	0.2036 (8)	0.0729 (4)	0.0279 (5)	−0.0044 (3)	−0.0229 (5)
S1	0.0553 (11)	0.0347 (8)	0.0547 (10)	0	0.0179 (8)	0
C9	0.043 (2)	0.038 (2)	0.047 (2)	0.001 (2)	0.0052 (18)	0.005 (2)
C11	0.058 (3)	0.085 (3)	0.053 (2)	0.009 (3)	0.005 (2)	−0.003 (3)
C4	0.044 (3)	0.106 (5)	0.042 (2)	0.005 (3)	0.006 (2)	−0.009 (3)
C1	0.044 (3)	0.053 (3)	0.037 (2)	0.001 (2)	0.012 (2)	0.002 (2)
C6	0.052 (3)	0.062 (3)	0.056 (3)	−0.002 (3)	0.007 (2)	0.016 (3)
N1	0.042 (2)	0.052 (2)	0.0439 (19)	0.0014 (17)	0.0134 (17)	0.0053 (16)
C5	0.063 (4)	0.084 (4)	0.053 (3)	0.010 (3)	0.003 (3)	0.014 (3)
C2	0.064 (4)	0.067 (3)	0.053 (3)	−0.002 (3)	0.017 (2)	0.011 (3)
C3	0.051 (3)	0.094 (4)	0.065 (3)	−0.019 (3)	0.017 (3)	−0.005 (3)
C7	0.051 (3)	0.060 (3)	0.043 (2)	0.006 (2)	0.017 (2)	0.011 (2)
C8	0.039 (3)	0.051 (3)	0.051 (3)	0.000 (2)	0.008 (2)	0.013 (2)
C10	0.057 (3)	0.035 (2)	0.073 (3)	−0.001 (2)	0.017 (3)	0.005 (2)

### Geometric parameters (Å, º)

**Table:**

Br1—C4	1.899 (5)	C1—C7	1.505 (6)
S1—C9^i^	1.724 (4)	C6—H6	0.93
S1—C9	1.724 (4)	C6—C5	1.383 (7)
C9—C8	1.442 (6)	N1—C7	1.468 (5)
C9—C10	1.356 (6)	N1—C8	1.265 (5)
C11—H11A	0.96	C5—H5	0.93
C11—H11B	0.96	C2—H2	0.93
C11—H11C	0.96	C2—C3	1.382 (7)
C11—C7	1.509 (7)	C3—H3	0.93
C4—C5	1.356 (7)	C7—H7	0.98
C4—C3	1.365 (7)	C8—H8	0.93
C1—C6	1.391 (6)	C10—C10^i^	1.405 (10)
C1—C2	1.383 (7)	C10—H10	0.93
			
C9—S1—C9^i^	91.4 (3)	C4—C5—H5	120.3
C8—C9—S1	121.6 (3)	C6—C5—H5	120.3
C10—C9—S1	111.2 (3)	C1—C2—H2	119
C10—C9—C8	127.1 (4)	C3—C2—C1	122.0 (5)
H11A—C11—H11B	109.5	C3—C2—H2	119
H11A—C11—H11C	109.5	C4—C3—C2	118.9 (5)
H11B—C11—H11C	109.5	C4—C3—H3	120.6
C7—C11—H11A	109.5	C2—C3—H3	120.6
C7—C11—H11B	109.5	C11—C7—H7	108.5
C7—C11—H11C	109.5	C1—C7—C11	113.2 (4)
C5—C4—Br1	119.5 (4)	C1—C7—H7	108.5
C5—C4—C3	121.3 (5)	N1—C7—C11	109.9 (4)
C3—C4—Br1	119.2 (4)	N1—C7—C1	108.2 (3)
C6—C1—C7	122.2 (4)	N1—C7—H7	108.5
C2—C1—C6	116.9 (4)	C9—C8—H8	117.9
C2—C1—C7	120.9 (4)	N1—C8—C9	124.1 (4)
C1—C6—H6	119.3	N1—C8—H8	117.9
C5—C6—C1	121.3 (5)	C9—C10—C10^i^	113.1 (3)
C5—C6—H6	119.3	C9—C10—H10	123.5
C8—N1—C7	116.7 (3)	C10^i^—C10—H10	123.5
C4—C5—C6	119.5 (5)		
			
Br1—C4—C5—C6	179.2 (4)	C2—C1—C6—C5	0.2 (7)
Br1—C4—C3—C2	−179.2 (4)	C2—C1—C7—C11	−122.6 (5)
S1—C9—C8—N1	4.2 (6)	C2—C1—C7—N1	115.3 (4)
S1—C9—C10—C10^i^	1.2 (7)	C3—C4—C5—C6	−2.3 (8)
C9^i^—S1—C9—C8	−177.3 (4)	C7—C1—C6—C5	−179.6 (4)
C9^i^—S1—C9—C10	−0.4 (3)	C7—C1—C2—C3	179.7 (4)
C1—C6—C5—C4	1.0 (7)	C7—N1—C8—C9	176.9 (4)
C1—C2—C3—C4	−1.0 (7)	C8—C9—C10—C10^i^	177.9 (5)
C6—C1—C2—C3	−0.2 (7)	C8—N1—C7—C11	131.9 (4)
C6—C1—C7—C11	57.3 (5)	C8—N1—C7—C1	−104.0 (4)
C6—C1—C7—N1	−64.8 (5)	C10—C9—C8—N1	−172.2 (5)
C5—C4—C3—C2	2.3 (7)		

Symmetry code: (i) −*x*+1, *y*, −*z*+1.

## References

[bb1] Agilent (2013). *CrysAlis PRO* and *CrysAlis RED* Agilent Technologies Inc., Santa Clara, CA, USA.

[bb2] Bernès, S., Hernández-Téllez, G., Sharma, M., Portillo-Moreno, O. & Gutiérrez, R. (2013). *Acta Cryst.* E**69**, o1428. 10.1107/S1600536813021685 PMC3884388 24427060

[bb3] Espinosa Leija, A., Hernández, G., Cruz, S., Bernès, S. & Gutiérrez, R. (2009). *Acta Cryst.* E**65**, o1316. 10.1107/S1600536809017528 PMC2969804 21583173

[bb4] Farrugia, L. J. (2012). *J. Appl. Cryst.* **45**, 849–854.

[bb5] Flack, H. D. (1983). *Acta Cryst.* A**39**, 876–881.

[bb6] Kudyakova, Y. S., Burgart, Y. V., Slepukhin, P. A. & Saloutin, V. I. (2012). *Mendeleev Commun.* **22**, 284–286.

[bb7] Sheldrick, G. M. (2008). *Acta Cryst.* A**64**, 112–122. 10.1107/S0108767307043930 18156677

[bb8] Tanaka, K. & Toda, F. (2000). *Chem. Rev.* **100**, 1025–1074. 10.1021/cr940089p 11749257

